# Retropharyngeal abscess revealing a migrant foreign body complicated by mediastinitis: a case report

**DOI:** 10.11604/pamj.2014.19.125.5334

**Published:** 2014-10-03

**Authors:** Tarik Ziad, Youssef Rochdi, Othmane Benhoummad, Hassan Nouri, Lahcen Aderdour, Abdelaziz Raji

**Affiliations:** 1 Otolaryngology Head and Neck Surgery Department, Mohammed VI University hospital, Marrakesh, Morocco

**Keywords:** Retropharyngeal, mediastinitis, abscess

## Abstract

Pharyngeal foreign bodies are quite common. Their diagnosis is usually easy. The risk of complications including retropharyngeal abscess and mediastinitis is rare and it depends mainly on the nature of the foreign body and the period of the therapeutic management. The occurrence of these complications darkens the prognosis of this affection usually benign. We report a 21 years old patient, without any significant history, admitted to the emergency for a high painful dysphagia and impaired general condition with fever 20 days after trauma in the posterior pharyngeal wall following a meal. The radiological assessment including cervico-thoracic CT scan had objectified the presence of a metallic foreign body in the retropharyngeal space associated with a retropharyngeal abscess and aggravated by a mediastinitis following the migration of the foreign body to the chest. Biological markers of infection were very increased. The therapeutic management consisted of a surgical drainage of the collections by a cervicotomy with removal of the foreign body. The outcome was favorable clinically and biologically. Pharyngeal foreign bodies are common and favorable when the diagnosis and extraction are made on time. The occurrence of complications, especially retropharyngeal abscess and mediastinitis is rare and burdened with a high morbidity and mortality.

## Introduction

Pharyngeal foreign bodies are quite common. Their diagnosis is usually easy. The risk of loco-regional complications, including retropharyngeal abscess is rare and depends essentially on the nature of the foreign body and the delay of the therapeutic management. The retropharyngeal abscess may progress to mediastinitis, which is life-threatening to a patient who initially consulted for a benign prognosis disease. The recent observation of a case of unknown retropharyngeal foreign body gave us the opportunity to make a review of these rare complications.

## Patient and observation

A 21 years old patient with no individual pathological history was presented 20 days after a posterior pharyngeal wall trauma produced when taking a meal. The chief complains were a very painful dysphagia, impaired general condition with fever. The general examination revealed a temperature of 39.5°C, a blood pressure 130/70 mmHg, an impaired general condition with asthenia, anorexia and a non quantified weight loss. The examination of the oral cavity revealed a bulge in the posterior wall of the oropharynx. The rest of the examination was without particularity.

**Figure 1:**
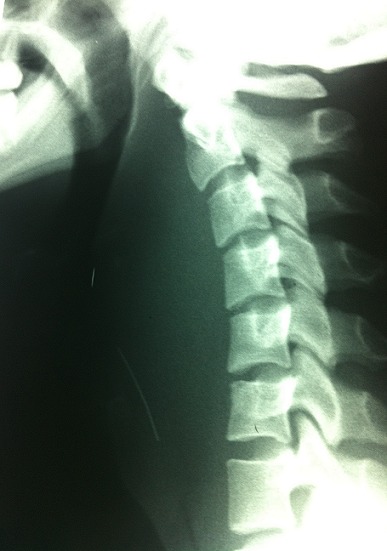
Radiography of the cervical spine showing an aspect of retropharyngeal collection and the presence of a radio opaque foreign body

**Figure 2:**
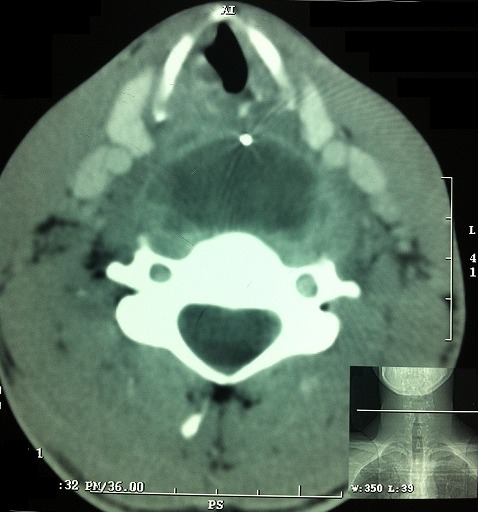
Cervical CT scan objectifying a retropharyngeal collection without discovertebral lysis and also confirming the presence of the foreign body

Radiography of the cervical spine showed an aspect of a retropharyngeal collection associated with a radio -opaque foreign body (Figure 1), and a cervical CT scan had objectified a retropharyngeal abscess without disco-vertebral lysis confirming the presence of the foreign body (Figure 2). An oral needle aspiration of the collection brought a frankly purulent fluid which was drained under general anesthesia. The patient had received a triple parenteral antibiotics therapy consisting of a combination of amoxicillin- clavulanic acid (1g every six hours), metronidazole (500 mg every eight hours) and aminoglycoside (160mg per day). After healing of the retropharyngeal infection on the tenth day of hospitalization, the patient was operated externally by a lateral-cervical incision to remove the foreign body, since the intraoperative X-ray intensifier control had not objectified it in the cervical region. A nasogastric tube was put in place to enable feeding and healing of the pharynx.

**Figure 3:**
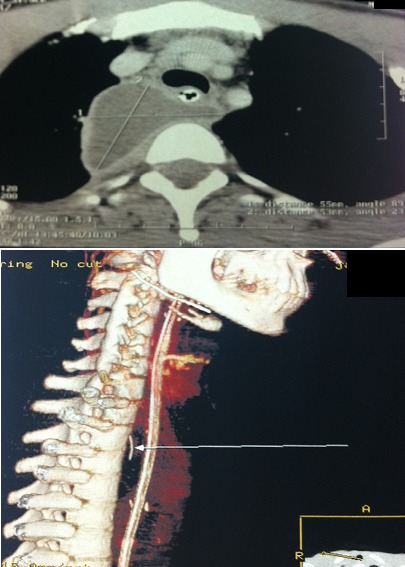
Cervicothoracic CT control objectifying migration of foreign body in the level of mediastinum with the presence of a mediastinal collection (A,B)

A cervicothoracic CT scan control was made and had objectified this time the migration of foreign body at the level of the mediastinum, associated with a mediastinal collection thus requiring an emergency reoperation in collaboration with a thoracic surgeon (Figure 3). The operation consisted of a mediastinal drainage through cervical route which also allowed the extraction of the foreign body, which was metallic, under endoscopic control (optic 0) through the drainage cavity and the implantation of drains in the medastinal and cervical region. The postoperative course was simple, cervical drain was removed on day 4 and mediastinal drain the 10th postoperative day and a tetanus serovaccination was performed. The patient left the hospital after improvement in the general condition and normalization of infectious.

## Discussion

Ingestion of foreign bodies was observed in 80% of cases of children in oral phase, usually between six months and three years [1, 2]. In adults, the ingestion of foreign bodies are mainly observed in patients with dental prosthesis, prisoners, psychotics or patients with mental retardation and alcoholics [3, 4]. In our case, the metallic foreign body was hidden in the meat which the patient accidentally ingested. The ingestion of a pharyngeal foreign body manifests clinically by a simple pharyngeal discomfort with a sudden onset during a meal, tenacious, localized, and often lateralized. It is most often a fish-bone or a bone fragment.

An attentive ENT examination with, indirect laryngoscopy with a tongue depressor then with a mirror allows the identification and removal in many cases. Fish bones are often planted in the tonsils and their removal is easy, by means of a clamp. General anesthesia may be necessary, especially for small children, in case of hypopharyngeal foreign body or if the patient is not cooperating, except when a large pharyngolaryngeal foreign body results in a aphagia with or without respiratory distress, thus imposing an emergency extraction. In cases where the foreign body remains neglected or ignored, the evolution is usually done towards the establishment of a retropharyngeal abscess in which the clinical diagnosis can be difficult. The clinical symptoms are variable and non specific. Infectious syndrome may be lacking in certain situations of immunosuppression [5]. A large number of differential diagnoses must then be discussed [6]. CT scan is an important contributing factor for diagnosis, but its limit is the fact that it can't differentiate a cellulitis from an abcess in the retropharyngeal space [7]. Plain radiography, in lateral view, is very specific when it shows air in the retropharyngeal space [6]. The implementation of radiological assessment should not delay medical support. In case of any suspicion of retropharyngeal abscess, the doctor must prescribe empiric antibiotic therapy that subsequently adapted to the results of antibiogram to prevent it from moving towards infection with a worse prognosis: mediastinitis. Acute mediastinitis is an infectious disease, life-threatening in many cases (20-40% mortality), that extends from the oropharynx, cervical or esophageal region [8]. The extension of the cervical infection to the mediastinum is due to the continuity of cervico-mediastinal fascia [9]. The infection may spread along three anatomical distribution routes: 1- the pretracheal space 2- perivascular space 3- the retropharyngeal space: the elective communication channel between the cervical region and the posterior mediastinum explaining 70% of mediastinitis [10]. The spread of infection can range from the skull base to the diaphragm and below, since there is continuity between the retropharyngeal, retroperitoneal and retro-esophageal space.

Early diagnosis of mediastinitis and therapeutic management are essential for optimal patient survival. The cervico-thoracic CT scan is essential for the diagnosis and follow-up. This infection of the mediastinum is extremely serious and suspected from clinical and radiological arguments. It must be confirmed by surgical exploration and the positive culture of per-operative microbiological samples [8]. The therapy is based on broad spectrum antibiotics, surgery, drainage and treatment of any organ failure. There is currently no standardized surgical therapeutic conduct. A minimally invasive surgical approach may be recommended when the diagnosis is made early and the thoracic drainage is effective. The clinical, laboratory and CT monitoring may indicate a thoracotomy [8].

## Conclusion

Pharyngeal foreign bodies are common and favorable when the diagnosis and extraction are made on time. The occurrence of complications, particularly retropharyngeal abscess and mediastinitis is rare and burdened with a high morbidity and mortality.
